# A trauma medical home, evaluating collaborative care for the older injured patient: study protocol for a randomized controlled trial

**DOI:** 10.1186/s13063-020-04582-x

**Published:** 2020-07-16

**Authors:** Damaris Ortiz, Ashley D. Meagher, Heidi Lindroth, Emma Holler, Sujuan Gao, Babar Khan, Sue Lasiter, Malaz Boustani, Ben Zarzaur

**Affiliations:** 1 Division of Acute Care Surgery, Department of Surgery, Indiana University School of Medicine, 545 Barnhill Dr., Emerson Hall, Indianapolis, IN 46202 USA; 2 Sidney & Lois Eskenazi Hospital Smith Level One Trauma Center, 720 Eskenazi Ave, Indianapolis, IN 46202 USA; 3 Indiana University Health, Methodist Hospital Level One Trauma Center, 1701 Senate Ave, Indianapolis, IN 46202 USA; 4 Division of Pulmonary, Critical Care, Sleep and Occupational Medicine, Department of Medicine, Indiana University School of Medicine, 1120 W. Michigan St., CL 260, Indianapolis, IN 46202 USA; 5 Center of Health Innovation and Implementation Science, Center for Translational Science and Innovation, 410 W. 10th St., Indianapolis, IN 46202 USA; 6 Indiana University Center of Aging Research, Regenstrief Institute, 1101 W. 10th St., Indianapolis, IN 46202 USA; 7 School of Nursing and Health Studies, University of Missouri, 2464 Charlotte St, Kansas City, MO 64108 USA; 8 Department of Surgery, University of Wisconsin School of Medicine and Public Health-Madison, 600 Highland Ave., Madison, WI 53792 USA

**Keywords:** Collaborative care, Trauma, Elderly, Older, Recovery, Non-neurologically injured

## Abstract

**Background:**

It is estimated that 55 million adults will be 65 years and older in the USA by 2020. These older adults are at increased risk for injury and their recovery is multi-faceted. A collaborative care model may improve psychological and functional outcomes of the non-neurologically impaired older trauma patient and reduce health care costs.

**Methods:**

This is a randomized controlled trial of 430 patients aged 50 and older who have suffered a non-neurologic injury and are admitted to a level one trauma center in Indianapolis, IN, or Madison, WI. Participants will be assigned to either the Trauma Medical Home (TMH) intervention or usual care. The TMH intervention is a collaborative care model that includes validated protocols addressing the multi-faceted needs of this population, with the help of care coordination software and a mobile office concept. The primary outcome is self-reported physical recovery at 6- and 12-month follow-up. Secondary outcomes include self-reported psychological recovery, acute health care utilization, and cost-effectiveness of the intervention at 6 and 12 months. The TMH collaborative care model will be delivered by a registered nurse care coordinator. The assessments will be completed by trained blinded research assistants.

**Discussion:**

The proposed study will evaluate a collaborative care model to help maximize psychological and functional recovery for non-neurologically injured older patients at four level one trauma centers in the Midwest.

**Trial registration:**

Clinical Trials. NCT03108820. Registered on 11 April 2017.

Protocol Version 6: Study # 1612690852. April 12, 2019.

Sponsor: Indiana University. Human subjects and IRB contact information: irb@iu.edu

Prospectively registered in the WHO ICTRP on 4 June 2017.

## Background

According to the US Census Bureau, the estimated population in the USA in 2018 aged 65 years old or greater is 52,443,114 people. From 2014 to 2060, the age group from 45 to 64 is expected to increase 19.8% and for those over 65, the increase is projected to be 112% [1]. Injury from motor vehicle crashes, falls, gunshot wounds, stabs, and natural or man-made disasters lead to 1.4 million hospitalizations per year in persons age 50 or older, and billions in health care costs [2, 3]. In addition to monetary costs, there is an emotional toll that affects patients and their loved ones, inhibiting their ability to fully recover. The prevalence of post-traumatic stress disorder (PTSD) in this vulnerable population of Americans varies from 40% at discharge to 23% at 1 year after injury. Similarly, up to 35% of trauma survivors demonstrate symptoms of depression [4–13].

For non-neurologically injured patients, those without traumatic brain injuries or spinal cord injuries, there is the potential for near full recovery from both physical and psychological disabilities and a return to a pre-injury level quality of life with intensive rehabilitation. However, with the significant fragmentation of care in our current health care system, these vulnerable patients are often left without adequate rehabilitation that results in residual physical and psychological disabilities with subsequent reduced quality of life long after injury [3, 5, 9, 10, 12]. Many leave the hospital without an established primary care provider or with a failure to communicate the recovery plans to an existing primary care provider [5, 13, 14]. According to the Institute of Medicine (IOM), post-injury care should be evidence-based, patient-centered, without fragmentation, and situated within a collaborative care model [15]. Recent randomized controlled trials have found that a collaborative care model was able to enhance quality of life, reduce care fragmentation, and improve psychiatric symptoms among traumatic brain or spinal cord injury survivors [16–19]. However, there has been no similar intervention targeting the non-neurologically injured patient. Collaborative care models have been successfully employed at our institution in older patients in the primary care setting, as demonstrated in the GRACE and IMPACT studies [20, 21]. This randomized controlled clinical trial aims to test the efficacy of a collaborative care model, named the Trauma Medical Home (TMH), in meeting the complex biopsychosocial recovery needs of injured older adults without neurologic injury. We hypothesize that individuals who receive the TMH intervention for 6 months will experience a better quality of life and lower health care utilization compared to individuals receiving only usual care. The TMH intervention will primarily aim to investigate the 6- and 12-month physical function, psychological recovery, and health care utilization in injured older adults. We hypothesize that coordinating care in a multi-disciplinary manner via the TMH intervention will improve physical and psychological outcomes and decrease health care utilization while increasing cost-effectiveness. The findings of this trial will provide trauma centers and trauma systems nationwide with a collaborative care model that can be replicated and implemented to improve the lives of older patients after injury.

## Methods/design

### Study population

This multi-center prospective randomized controlled trial aims to recruit 430 adults age 50 years or older. The study sites include Indiana University Health Methodist Hospital, Sidney & Lois Eskenazi Hospital, St. Vincent Hospital in Indianapolis, IN, and University of Wisconsin Health University Hospital in Madison, WI. Combined, these American College of Surgeons verified level one trauma centers evaluate and treat well over 10,000 injured patients annually. Indiana University Health-Methodist Hospital (MH) has 625 beds, with over 3000 yearly trauma admissions, and Sidney & Lois Eskenazi Hospital (EH) has 327 beds and admits nearly 2000 trauma patients yearly. St. Vincent Hospital (SV) has 616 beds and admits close to 3000 trauma patients, and University of Wisconsin (UW) has 505 beds and admits almost 3000 trauma patients annually. MH and UW are tertiary academic centers and receive trauma transfers from across the Midwest. EH is a public access county hospital primarily serving Marion County, IN, and it is the largest safety-net hospital in the state. SV is a large trauma and transplant center that services the greater Indianapolis metropolitan area.

The target sample size of 430 patients (215 per group) is powered to detect a significant effect of 0.325 standard deviation or larger with 80% power on our outcome measurements. This is anticipating 30% lost to follow-up or death at the 12-month assessment. This sample size estimation is based on previous literature utilizing the Short Performance Physical Battery (SPPB) score [12, 22–25]. Assuming a mean SPPB score of 6.0 (SD 2.5), a sample size of 150 is needed to obtain 80% power and to detect a change score of 0.81, using a two-sample *t* test at 0.05.

### Inclusion/exclusion criteria

This study will be conducted in a population of English-speaking injured adults, age 50 and over, with an injury severity score (ISS) of 9 or greater that reside within 50 miles of the admitting trauma center and have access to a telephone. Eligible individuals must be able to provide informed consent or have a legally authorized representative provide informed consent. Exclusion criteria are defined by the presence of significant head injury (defined as any intracranial blood on computed tomography scan of the head, or Glasgow Coma Scale score of less than 13), history of neurodegenerative disease (including dementia, Alzheimer disease, or Parkinson disease), spinal cord injury with persistent neurologic deficit at the time of enrollment, a burn that involves greater than 10% of total body surface area, pregnancy (determined by a urine pregnancy test), incarceration, acute stroke upon admission or while hospitalized, malignancy with less than 1 year of life expectancy, recent alcohol or drug use disorder (within the past 6 months) as determined from the medical record and/or the Drug Abuse Screening Test or the Alcohol Use Disorders Screening Test C (AUDIT-C), or sensory impairment that would preclude active participation with study assessments and/or communications.

### Ethics and informed consent

The Indiana University Institutional Review Board approved this randomized controlled trial (IRB# 1612690852) and is registered on ClinicalTrials.gov (NCT03108820). All eligible participants will be approached prior to discharge for enrollment into the study. Informed consent will be obtained from the patient or legally authorized representative by a trained Research Assistant (RA) or Research Coordinator. After verbal explanation of the study, trial participants or their proxy will be provided with a printed consent document and HIPAA release form. Participants and their family members will have all questions answered during the consenting process. Any concern about the patient’s capacity to provide informed consent is brought to the attention of a trauma physician for formal assessment. Risks of trial participation are expected to be exceedingly rare. Participants or their surrogates can choose to withdraw from the study at any time, for any reason. Copies of the signed consent documents will be provided to the trial participant or surrogate upon completion. All research personnel will be IRB-approved and appropriately trained on confidentiality and enrollment procedures. Eligible and enrolled participant data will be collected, shared, and maintained in accordance with the Indiana University IRB and HIPAA guidelines. Only IRB-approved study team members will have access to the collected data and all consented participants will be assigned a unique study identifier upon enrollment. The data will be stored in a secure REDCap (Research Electronic Data Capture) database on a password-protected IU server. REDCap was specifically developed around HIPAA security guidelines. Any patient data used for ancillary studies will require a signed consent from every participant if the data collection/request is not covered in the original informed consent process. Any ancillary studies will go through IRB review.

### Adverse events

Adverse events will be reported to the PI, statistician, and DSMB as well as the IRB and will be reviewed for appropriate action per IU IRB policy. Adverse event forms will be used to formally document such occurrences. Research staff routinely monitor the EMR for any new ED visits or hospitalizations and these are recorded on an electronic spreadsheet. Deaths are also reported either by a notification in the EMR or from family members when they are called to schedule Outcomes Assessments. These reports are provided to the DSMB at each meeting. Other adverse events would be provided by spontaneous reports from patients or their families.

### Study design

This is a prospective, single-blind, multi-center, randomized controlled clinical trial utilizing a computer-generated stratified randomization scheme into the TMH intervention or control group (usual care) in a 1:1 manner. All participants will receive usual care. Those randomized to the TMH intervention will *also* receive the collaborative care (TMH) intervention. Outcome assessments will be performed at baseline, 6 months, and 12 months on each group by research staff blinded to treatment assignment, and mixed-effects models will be used to determine superiority of the TMH intervention on physical, psychological, and health care utilization outcomes.

### Description of intervention

The primary intervention of this study is the TMH collaborative care model, which will use evidence-based protocols and a team-based approach to address the specific rehabilitation needs of the non-neurologically injured older adult. Upon enrollment and informed consent by an RA/RC, participants will complete a baseline assessment. They will then be randomized to usual care or the intervention by an unblinded research staff member. If randomized to intervention, the patient still receives usual care. They will also set up a time for the First Home Visit by a trained collaborative care nurse (CCN) shortly after discharge from acute care. Information collected at the First Home Visit will trigger the use of specific evidence-based care protocols and the interdisciplinary team (consisting of a trauma surgeon, a health services researcher, geriatrician, psychologist, a critical care physician, and a CCN) will work together to develop a personalized plan of care that will be implemented over the 6-month intervention period. The CCN will take the care plan back to the patient and work with them throughout the intervention on a mutually agreed upon schedule. Using a mobile office concept, the patient or caregiver can decide the most convenient follow-up method, whether it be meeting at their home, at a physician office, place in the community, or via phone interaction. The Healthy Aging Brain Care Monitor (HABC-M) will be used throughout the course of the intervention to dynamically inform the activation and deactivation of care protocols to best suit patients’ changing needs. The intervention plan is summarized in Table 1. There are five phases to the intervention, followed by Outcomes Assessments at 6 months and 12 months. The intervention is intentionally timed during the most vulnerable phases of the conceptual recovery model, as summarized in Table 2.

**Table 1:** Intervention Plan

Intervention phase	Timeline	Description
Baseline assessment and usual care	In the hospital, after obtaining informed consent, prior to or shortly after discharge.	Obtain baseline functional and psychological assessments using the following: • Short Physical Performance Battery (SPPB), • Patient Health Questionnaire-9 (PHQ-9), • Generalized Anxiety Disorder Scale (GAD-7), • Medical Outcome Study Short Form (SF-36). See description of Usual Care.
First home visit (TMH intervention starts)	After hospital discharge, 0–1 month post-injury.	Obtain physical, cognitive, and psychological assessments, social and community needs assessment for patient and caregiver, and a thorough medication reconciliation. Review of medical appointments. Use of Healthy Aging Brain Care Monitor (HABC-M) to document symptoms and trigger treatment protocols.
Plan of care development	From start of first home visit to end of second home visit.	Emphasis on coordination of care between primary and specialty services. Document and finalize individualized care plan.
Second home visit	Within 1–2 weeks of the first home visit.	Implementation of individualized care plans and treatment protocols, dissemination of educational materials, and connection to in-home and community services.
6-month interaction period	From first home visit to end of 6 months.	Bi-weekly contact with patient and caregiver at minimum, continue to address identified needs and reinforce treatment protocols, revising as needed. At end of 6 months, transition care to primary care provider.

**Table 2:** Conceptual recovery model

TMH intervention timing with recovery	
Phase of recovery	Intervention
Acute (0–1 month post-injury)	Initial case review. Initial home visit, plan of care development.
Recovery (2–4 months post-injury)	Interaction period. Follow-up home visit. Initiation of care protocols. Implementation of recovery care plan, coordination of rehabilitation, and follow-up care.
Rehabilitation (5–6 months post-injury)	Continued interaction via face-to-face, telephone, or electronic means. Monitor and revise recovery care plan as needed. Transition of care plans to primary care provider at 6-months post-injury.
Stable (6–12 months post-injury)	Outcome assessments will be administered by blinded research personnel.

### Usual care

Usual care refers to the current practice of discharging an injured patient with follow-up care provided at the discretion of various specialists, depending on injury types, without a formal plan for coordination of specialists. There is also no standardized format to assess psychological well-being after discharge. Upon discharge from acute care, patients are provided with a discharge summary and discharge instructions describing the patient’s hospital course, injuries, new diagnoses, medications with dosages, and post-injury rehabilitation plan. Patients may also receive educational materials on caregiver coping mechanisms and legal and financial advice if requested. Patients are encouraged to follow-up with their primary care provider or injury specialist for any continuing care needs. All usual concomitant care is permitted as long as the patient does not meet any exclusion criteria. There is no formalized system of coordinating appointments or care plans between specialist teams and the primary care provider. Patients in the usual care arm will receive no further interventions.

### Randomization and blinding

The RC/RA will enroll participants after informed consent has been obtained. A separate unblinded research staff member will randomize participants to the intervention or usual care based on a computer-generated allocation sequence and notify the CCN of randomization to intervention. Randomization is stratified by injury severity score (ISS), mechanism of injury, and recruitment site. Random block sizes of 2 and 4 are used within each strata.

The RC and RA performing outcomes assessments will be blinded to the treatment assignment. A separate unblinded RA will randomize patients after the RC and RA have completed eligibility confirmation and baseline assessments. The CCN is unblinded and is notified of the patients assigned to the intervention arm. Trial participants and care providers must be unblinded due to the nature of the intervention.

### Assessments and outcomes

The primary outcome of the study is to assess the ability of the TMH intervention to improve physical recovery of injured older adults, as measured by the Short Physical Performance Battery (SPBB) and the Physical Component Summary (PCS) of the Medical Outcomes Study 36 short form (SF-36). Secondary outcomes of the study are to assess the ability of the TMH intervention to improve psychological and functional outcomes, as well as patient-reported quality of life as measured through the Medical Study Short Form 36 (SF-36), Activities of Daily Living (Katz Index of Independence in Activities of Daily Living and Lawton Instrumental Activities of Daily Living scales), Patient Health Questionnaire-9 (PHQ-9), and Generalized Anxiety Disorder Scale (GAD-7). Outcome assessments will be administered by trained RCs who are blinded to the intervention at enrollment (baseline), 6 months post-injury, and 12 months post-injury. The method of aggregation will include the proportion of change in scores from the baseline assessment to the 6- and 12-month follow-up in order to evaluate the effect of the TMH intervention on the stable phase of recovery as compared to those who receive only usual care.

Additionally, this study aims to examine the effect of the TMH intervention on health care utilization and cost. This outcome will be examined by collecting electronic medical record information on emergency department, hospital admission, and other health care-related encounters. This data will allow us to enumerate emergency department visits and hospitalizations within 6 months of discharge as well as the diagnoses associated with each utilization episode. We expect that the TMH intervention will decrease health care cost and utilization. The method of aggregation will include a comparison of median health care facility visits and cost-effectiveness ratios at baseline, 6 months, and 12 months between the TMH intervention group and the Usual Care control group.

### Data collection

Demographic and medical data will be collected after enrollment, including the participant’s age, race, gender, years of education completed, income bracket, height, weight, body mass index, blood pressure, and Charlson Comorbidity Index [26]. We will also utilize the trauma registries maintained at the level one trauma centers to obtain detailed demographics, injury type, injury location, injury severity, treatment, and complication information.

We will use the Eskenazi Health financial record system and the local data-warehouse of Indiana University Health (IUH) and IUH Physician Group (IUHP) to capture data needed to determine health care utilization. The IUHP data warehouse includes detailed administrative, billing and hospital records of all patients seen within the IUH system, which encompasses nearly 60% of health care in the state of Indiana. Furthermore, we will use the data from the Indiana Network for Patient Care (INPC) to capture any health care utilization outside of the IUH system. INPC is the primary health information exchange in the state of Indiana and it provides data for acute care services from all of the health care systems within the state. We will determine the number of emergency department visits and the number of re-hospitalizations throughout the entire study period as well as the diagnoses associated with each utilization episode. For patients admitted to UW, we will use all claims data from the Wisconsin Health Information Organization (WHIO) to determine health care utilization in the follow-up period. The WHIO all claims database captures 75% of hospital and emergency department visit in the state of Wisconsin.

All baseline assessments will be completed in the participant’s hospital room by trained and blinded RC/RA. Follow-up assessments will be completed by the RC/RA’s at a mutually agreed upon location with the participant or their proxy. Assessments will be entered into a REDCap (Research Electronic Data Capture) database, an electronic data capture tool hosted at Indiana University Clinical Translational Science Institute. REDCap is a secure, web-based application designed to support data capture for research studies, providing validated data entry, audit for tracking data manipulation and export procedures, automated export procedures, and procedures for importing data from external sources. Frequency, timing of contacts, and the intervention offered in the group receiving TMH will be tracked using the HABC Trauma Medical Home software which offers quantitative measures of intervention intensity in the TMH group. All of the cognitive, physical, psychological, and quality of life outcome measures will be assessed at baseline (hospital discharge) and at 6- and 12-month follow-up. While some assessments are recorded on paper, all are directly entered into the electronic system in REDCap. To monitor data quality and completeness, a separate RA or RC than the assessor reviews the data entered within a week of entry.

### Description of study instruments

The following four instruments will be used to assess participant outcomes.

### Short Physical Performance Battery (SPPB)

Physical recovery effects will be assessed via the SPPB, a validated objective assessment [14, 27–30]. The SPPB yields a performance score of 0–12: 0–4 is poor, 5–7 intermediate, 8–12 good. Based on previous studies in similar patient populations, the expected scores on the SPPB are 6.0 (SD 2.5) (at baseline), 7.5 (SD 2.5) at 6 months, and 8 (SD 2.5) at 12 months. A difference of more than 1.3 would be considered clinically significant between the control and intervention groups.

### Medical Outcome Study Short Form (SF-36)

Non-neurologically injured patient’s health-related quality of life will be assessed using the Medical Outcome Study Short Form (SF-36). This scale has eight components (physical functioning, role-physical, bodily pain, general health, vitality, social functioning, role-emotional, and mental health) that are aggregated into a Physical Component Summary (PCS) and a Mental Component Summary (MCS). Expected scores on the PCS of the SF-36 are 40 (SD 5) at baseline, 49 (SD 5) at 6 months, and 51 (SD 5) at 12 months. A difference of more than 2 points would be considered clinically significant for both the PCS and MCS [31–33].

### Patient Health Questionnaire-9 (PHQ-9) and Generalized Anxiety Disorder Scale (GAD-7)

The PHQ-9 [34, 35] is a nine-item depression scale with a total score from 0 to 27 and the GAD-7 [36] is a seven-item anxiety scale with a total score from 0 to 21. Both of these scales are derived from the Patient Health Questionnaire and have good internal consistency, test-retest reliability, and convergent, construct, criterion, procedural, and factorial validity for the diagnosis of major depression and general anxiety disorder [35, 37]. A change of 2 points is considered statistically significant.

### Analyses

Participant baseline characteristics will be compared using analysis of covariance (ANCOVA) for continuous variables and the Cochran-Mantel-Hansel statistic for categorical variables while controlling for recruitment sites to verify the comparability of the randomized groups. Data distributions and frequencies will be examined. Alternative approaches such as transformations or nonparametric methods will be used if continuous data does not follow normal distributions. In the case of zero or small cell sizes, we will adopt exact inference procedures. SAS 9.4 (SAS Institute, Cary, NC) will be used for all analyses and significance noted at *p* ≤ 0.05.

The primary aim is to evaluate the ability of the TMH intervention to improve the physical recovery of non-neurologically injured patients. Mixed effect models will be used to evaluate the physical function scores (SPPB, PCS on the SF-36) collected at baseline, 6 months, and 12 months. Independent variables will include the assigned group, time of evaluation, and a group and time interaction while adjusting for stratification variables (recruitment site, injury severity, injury type) and baseline covariates that are found to be significantly different between the intervention and usual care group. To account for the potential correlations between the physical function scores (SPPB, PCS on the SF-36) within individuals over time, an unstructured variance-covariance matrix will be used in the mixed effects model. Following significant interactions between group and time, post hoc comparisons will be conducted at each follow-up time to determine the point at which a group difference is detectable. A maximum likelihood approach will be used to generate parameter estimates and inferences, which are robust under several missing data mechanisms [38].

The second aim is to evaluate the ability of the TMH intervention to improve the psychological recovery of the non-neurologically injured patient. The mixed effect modeling approach outlined for our primary outcome will be applied to investigate our secondary outcome of psychological recovery measured with PHQ-9, GAD-7, and MCS on SF-36 scores collected at baseline, 6 months, and 12-months.

The third aim is to evaluate the ability of the TMH intervention to reduce health care costs associated with increased health care utilization and to examine the cost-effectiveness of the TMH intervention relative to usual care in the non-neurologically injured patient in terms of economic value and costs. To examine the ability of the TMH intervention to reduce health care utilization, a Cox’s proportional hazard model will be used. Event time will be censored at 12 months for those participants who are followed to 12 months without experiencing any outcome event. Participants who died or were lost to follow-up will have their observation time censored at time of death or data of last contact. The outcome is measured as time from enrollment to emergency department visits and hospital readmissions. Group assignment, time of evaluation, and baseline covariates found to differ significantly between groups will be included in the model.

The economic value and costs associated with the TMH intervention will be evaluated using an established method from a Medicare payment perspective. The cost-effectiveness of the intervention is measured by increments in the cost summary (both health care and non-health care-related expenses) and effect (return to function measured by SF-36). Foregone economic opportunities are captured by the recovery time in dollar value. Multivariate regression models will examine total health care costs in the 12-month post-index period. These data will inform the cost-effectiveness ratio, which is the difference in intervention costs of the treatment arms, divided by the difference in effectiveness between groups. Sensitivity analysis and bootstrapping will be completed to ensure robustness of findings.

### Data safety monitoring board and data safety

The trial will be audited every 6 months by the Data Safety and Monitoring Board (DSMB) and reviewed annually by the Indiana University IRB through the continuing review. The DSMB meets every 6 months and is composed of three members: a safety officer, an expert trauma surgeon, and a biostatistician. This team is separate from IU and has no competing interests. The safety officer is a physician researcher with extensive experience in intervention studies. This individual reviews reports generated by the study manager and biostatistician to determine if additional action is necessary. This could include corrective action, an ad hoc review, a stopping rule violation, or the need to communicate out of range data to the provider or patient. Table 3 illustrates the frequency and type of data reviewed. All reports will be provided to the IRB at the time of continuing review. Any decision to terminate the trial early will be determined by the IU IRB. Additional information regarding the DSMB is readily available by contacting the study PI.

**Table 3:** Data review schedule

Data Type	Frequency of review		
	Each occurrence	Q 6 months	Annual
Participant accrual (adherence to inclusion/exclusion); drop-out rates; randomization		X	
Adverse event rates (injuries)	X	X	
Participant complaints		X	
Compliance to interventions		X	
Protocol violations/noncompliance	X	X	
Out of range data		X	
Risk-benefit ratio assessment			X
Stopping rules report			X

The Research Operations Committee meets on a weekly basis and is composed of the PI, trauma surgeons, the RC, RA’s, an analyst, and a geriatrician. Weekly enrollments are discussed as well as specific concerns pertaining to study operations at the various sites. Any potential amendments to the protocol are discussed at this meeting, including unblinding of staff. Amendment proposals are then submitted to the IRB for approval. Upon IRB approval, the PI will inform the relevant parties through formal communications. This committee also oversees data analysis and plans for dissemination of results. This includes plans for presentation of results at national meetings, as well as abstract and manuscript writing for journal publications.

### Special considerations

#### Missing data

Two different forms of missing data are anticipated with this trial; those lost to follow-up and those lost to death. The usual care group may experience a higher rate of lost follow-up when compared to the intervention group due to frequent contacts between the study team and the participants in the intervention group. In anticipation of these missing data, we will use a mixed effect model approach, which is robust under the missing at random assumption, i.e., the probability of missing is unrelated to the missing outcomes. Baseline characteristics of patients with missing outcomes will be compared to detect potential violations to the missing at random assumption. Sensitivity analyses using various methods of imputation or a full parametric likelihood approach assuming various patterns of missing data will be performed if the missing at random assumption is violated. The intention to treat principle will be used in all models.

#### Recruitment and retention

The trauma service census will be screened daily by the RC for eligible participants. Eligible participants will be approached prior to hospital discharge for study enrollment. Gift card incentives will be utilized at the completion of the 6- and 12-month follow-up visits to encourage participation. The CCN’s mobile office concept also helps to improve retention in the intervention group, as the patient and/or caregiver identifies the most convenient location for follow-up meetings. This could be at home, at the clinic or hospital, or designated areas in the community. The TMH validated care protocols address various issues such as medication adherence and compliance with physical therapy. Additionally, the interactive phase of the intervention stresses the importance of frequent communication between the patient and caregiver and the care coordinator, which can be face-to-face, by telephone, or by electronic means. Patients must have access to a telephone to be participants in the study. Study retention will be periodically measured. If retention drops below 80%, the study staff will attempt to follow-up with the study participants to troubleshoot issues and provide coaching in order to prevent more losses.

## Discussion

Optimizing outcomes in older adults following a traumatic event is key to avoiding declines in both physiologic and psychologic function. Collaborative care for patients recovering from a trauma-related neurological injury has been shown to improve patient outcomes [16–19]. Nonetheless, it is unclear if older adults with a non-neurologic traumatic injury will benefit. This randomized controlled trial aims to determine whether a collaborative care model improves physical and psychological outcomes while reducing health care utilization and cost in older adults recovering from a non-neurologic traumatic injury. Outcome measurements at baseline, 6 months, and 12 months will allow for assessment of the study’s long-term impact on quality of life and return to functional status after traumatic injury.

A major strength of this study is the use of a customizable collaborative care intervention for recovery for the older injured patient. Technology enables the intervention to be tailored to each participant’s specific needs using the 32-item process measurement tool, the HABC-M [39], making the assessment and intervention process dynamic. The ability to quickly adapt the intervention is highly beneficial given the frequently changing needs of this population. In addition, the mobile office concept allows the intervention to take place at locations convenient for the patient, decreasing the need for patient travel, and reducing potential barriers to care. We also anticipate several limitations. Though it is a multi-center study, it represents two Midwestern cities in the USA and may have limited generalizability to other regions of the country. Second, the study does not account for existing protocols in primary care for specialist coordination and follow-up so we cannot rule out the possibility of cross-arm contamination.

Older injured adults are a fast-growing vulnerable population with complex medical needs whose outcomes can be negatively impacted by health care fragmentation and lack of psychosocial support. The current health care system is failing to adequately address the unique needs of these patients and alternative strategies are needed. In contrast to current usual care, the TMH intervention has the potential to restore older adults back to or close to their baseline level of functioning prior to injury. In addition to physical recovery this protocol also aims to address psychological sequelae of trauma, which is increasingly recognized as a significant morbidity after injury [12, 40]. The intervention acknowledges mental health as an important factor that influences the recovery process as part of the collaborative care model. The Trauma Medical Home has the potential to reduce health care delivery fragmentation, reduce health care utilization, and improve physical and psychological outcomes. Ultimately, the success of this study could provide a scalable recovery model to remediate current treatment strategies and ameliorate this gap in care in order to help older injury survivors achieve the best possible quality of life.

## Trial status

Enrolling. Date recruitment began: 10/2017. Approximate date recruitment will be completed: 1/31/2021. Protocol version 6. Study # 1612690852. April 12, 2019. A chronology of the protocol revisions with dates can be found on the Spirit Checklist supplemental document.

## Supplementary information

**Additional file 1.** SPIRIT 2013 Checklist: Recommended items to address in a clinical trial protocol and related documents.

**Additional file 2.** Indiana University informed consent statement for elderly trauma medical home.

## Data Availability

Not applicable.

## Abbreviations

AUDIT-C: Alcohol Use Disorders Screening Test C
CCN: Collaborative Care Nurse
DSMB: Data and Safety Monitoring Board
GAD-7: Generalized Anxiety Disorder Assessment-7
HIPAA: Health Insurance Portability and Accountability Act
HABC-M: Healthy Aging Brain Care Monitor
IRB: Institutional Review Board
IUH: Indiana University Health
IUHP: Indiana University Health Physicians
MCS: Mental Component Summary
PTSD: Post traumatic stress disorder
RA: Research Assistant
RC: Research Coordinator
REDCap: Research Electronic Data Capture
SD: Standard deviation
SF-36: Medical Outcome Study Short Form
SPPB: Short Physical Performance Battery
TMH: Trauma Medical Home
PCS: Physical Component Summary
PHQ-9: Patient Health Questionnaire-9
PI: Principal Investigator
