# Supplementary material for: A trauma medical home, evaluating collaborative care for the older injured patient: study protocol for a randomized controlled trial
Source: Trials. 2020 Jul 16;21:655. doi: 10.1186/s13063-020-04582-x (PMC7364470; doi:10.1186/s13063-020-04582-x)
Supplement: Supplementary file 1 — Additional file 1. SPIRIT 2013 Checklist: Recommended items to address in a clinical trial protocol and related documents. [file 13063_2020_4582_MOESM1_ESM.doc]

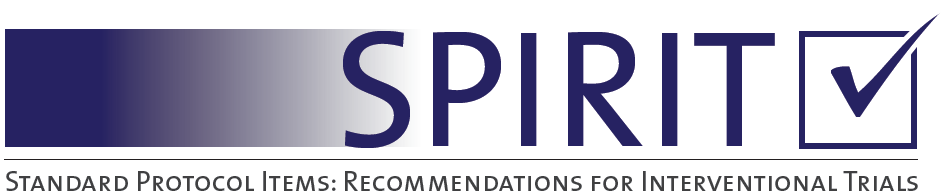


SPIRIT 2013 Checklist: Recommended items to address in a clinical trial protocol and related documents*

| Section/item | ItemNo | Description |
| --- | --- | --- |
| **Administrative information** | | |
| Title | 1 | Descriptive title identifying the study design, population, interventions, and, if applicable, trial acronym.  A Trauma Medical Home, Evaluating Collaborative Care for the Older Injured Patient: Study Protocol for a Randomized Controlled Trial. Addressed on manuscript page 1. |
| Trial registration | 2a | Trial identifier and registry name. If not yet registered, name of intended registry.  Trial registration: ClinicalTrials.gov  NCT03108820  NIH. Addressed on manuscript page 1. |
|  | 2b | All items from the World Health Organization Trial Registration Data Set.   1. **Primary Registry and Trial Identifying Number** ClinicalTrials.gov, NCT03108820 2. **Date of Registration in Primary Registry** 11 April, 2017 3. **Secondary Identifying Numbers** 1612690852   1R01AG052493-01A1   1. **Source(s) of Monetary or Material Support** National Institutes of Health 2. **Primary Sponsor** Indiana University 3. **Secondary Sponsor(s)** Not applicable. 4. **Contact for Public Queries** Emma Holler, [emma.holler@eskenazihealth.edu](mailto:emma.holler@eskenazihealth.edu), (317) 880-5033,   720 Eskenazi Ave, Indianapolis, IN 46202   1. **Contact for Scientific Queries** Principal Investigator: Ben Zarzaur MD, MPH, [zarzaur@surgery.wisc.edu](mailto:zarzaur@surgery.wisc.edu), 600 Highland Ave, Madison, WI 53792, (608) 263-2284 2. **Public Title** A Trauma Medical Home, Evaluating Collaborative Care for the Older Injured Patient: Study Protocol for a Randomized Controlled Trial. 3. **Scientific Title** Collaborative Care for the Older Injured Patient: A Trauma Medical Home 4. **Countries of Recruitment** The United States of America 5. **Health Condition(s) or Problem(s) Studied** Physical and psychological recovery after non-neurologic traumatic injury. 6. **Intervention(s)** Trauma Medical Home Intervention: Collaborative care designed to address physical, psychological, and social aspects of recovery from discharge to 6 months after injury.   Usual Care(Control Arm): Patients receive discharge instructions for wound care and follow-up appointments with no additional coordination of care. Financial 3ounselling and other social services may be available upon request.   1. **Key Inclusion and Exclusion Criteria** Ages eligible for study: 50 years old or older   Sexes eligible for study: both  Accepts healthy volunteers: no  Inclusion Criteria: English-speaking, ISS≥9, lives 50 miles within admitting trauma center, non-neurologically injured, has access to a telephone, able to give consent or has a surrogate to give consent.  Exclusion Criteria: Neurologic injury or disease, pregnant, incarcerated, malignancy with less than 1-year life expectancy, recent drug or alcohol use disorder, sensory impairment that will preclude assessments, burn affecting >10% TBSA.   1. **Study Type**   Type of study: Interventional  Study design including:  Method of allocation: Randomized. Computer-generated allocation sequence, stratified by ISS, mechanism of injury, and recruitment site. Allocation concealment mechanism: records kept by an unblinded research staff member.  Masking: Double (Investigator, Outcomes Assessor)  Assignment: Parallel  Purpose: Supportive Care  Phase: Not applicable.   1. **Date of First Enrollment** October 2017 2. **Sample Size**   Number of participants that the trial plans to enrol in total: 430  Number of participants that the trial has enrolled: 200   1. **Recruitment Status** Recruiting: participants are currently being recruited and enrolled   **Primary Outcome(s)** 1. Change in Self-Reported Physical Recovery, at 6- and 12- months after injury. As determined using the Physical Component Score of Short Form (SF-36). 2. Change in Physical Recovery, at 6- and 12- months after injury. As determined using the Short Physical Performance Battery (SPPB).  .**Key Secondary Outcomes**  1. Change in Self-Reported Psychological Recovery, at 6- and 12- months after injury. As determined using the Mental Component Score of SF-36, and the Patient Health Questionnaire (PHQ-9) for depression and anxiety scores  2. Change in Healthcare Utilization, at 6- and 12- months after injury. As determined by hospital readmission.  3. Cost effectiveness at 6- and 12- months after injury. As determined using the cost-effectiveness ratio.  .**Ethics Review**  Status: Approved on 18 February, 2017, reviewed annually.  Name and contact details of Ethics committee: Indiana University Institutional Review Board, [irb@iu.edu](mailto:irb@iu.edu)  Data Safety Monitoring Board: meets every six months. Chris Cox, Marcel Salive, Lona Mody, Oscar Guillamondegui. Main contact: marcel.salive@nih.gov   1. **Completion date** Projected date of study completion: December 2025 2. **Summary Results** No results or publications to date. 3. **IPD sharing statement**   Plan to share IPD: No |
| Protocol version | 3 | Date and version identifier. Addressed on manuscript page 14.  Issue date 12 April 2019. Protocol V6, Amendment number 5.  Authors EH, BZ  Revision Chronology:  V1: 2016-Dec-5 Original  V2: 2017-Oct-23 Amendment 01. Amendment to add Katz/Lawton scales, add exclusion criteria due to inability to complete questionnaires due to extreme hearing loss and recent substance or alcohol abuse.  V3: 2018-Apr-26 Amendment 02. Amendment to edit exclusion criteria to exclude patients who have an acute stroke upon admission or a burn affecting >10%TBSA.  V4: 2018-Aug-20 Amendment 03. Amendment to allow study team to mail outcome assessment forms to participants with return postage-paid envelope if unable to contact them via phone.  V5: 2019-Jan-11 Amendment 04. Amendment to allow RC’s to finish enrollment after hospital discharge if patient expressed interest in the study but were unable to get consented before they left the hospital.  V6: 2019-Apr-3 Amendment 05. Amendment to change the IU site PI to Ashley Meagher, MD, MPH. |
| Funding | 4 | Sources and types of financial, material, and other support.  HL is supported by T32: NHBLI 5T32HL091816-07, the Trauma Medical Home is funded by NIH Grant 1R01AG052493-01A1. Addressed on manuscript page 15. |
| Roles and responsibilities | 5a | Names, affiliations, and roles of protocol contributors. Addressed on manuscript title page.  Damaris Ortiz, MD^1,2,*^ (damaorti@iu.edu)  Ashley D Meagher, MD MPH^1,3^ (ashmeagh@iu.edu)  Heidi Lindroth, PhD RN^4,5,6^ ([hlindrot@iu.edu](mailto:hlindrot@iu.edu))  Emma Holler, BS CCRP^2^ (emma.holler@eskenazihealth.edu)  Babar Khan MD MS^4,5,6^ (bakhan@iu.edu)  Sue Lasiter PhD RN^8^ (lasiterr@umkc.edu)  Malaz Boustani, MD MPH^5,6^ (mboustan@iu.edu)  Ben Zarzaur, MD MPH FACS^7^ (zarzaur@surgery.wisc.edu)   1. Division of Acute Care Surgery, Department of Surgery, Indiana University School of Medicine, 545 Barnhill Dr., Emerson Hall, Indianapolis, IN 46202 2. Sidney & Lois Eskenazi Hospital Smith Level One Trauma Center, 720 Eskenazi Ave, Indianapolis, IN 46202 3. Indiana University Health, Methodist Hospital Level One Trauma Center, 1701 Senate Ave, Indianapolis, IN 46202 4. Division of Pulmonary, Critical Care, Sleep and Occupational Medicine, Department of Medicine, Indiana University School of Medicine, 1120 W. Michigan St., CL 260, Indianapolis, IN 46202 5. Center of Health Innovation and Implementation Science, Center for Translational Science and Innovation, 410 W. 10^th^ St., Indianapolis, IN 46202 6. Indiana University Center of Aging Research, Regenstrief Institute, 1101 W. 10^th^ St., Indianapolis, IN 46202 7. Department of Surgery, University of Wisconsin School of Medicine and Public Health-Madison, 600 Highland Ave., Madison, WI 53792 8. University of Missouri, Kansas City, School of Nursing and Health Studies, University of Missouri, 2464 Charlotte St, Kansas City, MO 64108   * Corresponding Author |
|  | 5b | BZ and MB developed the theoretical framework for the study. BZ, MB, BK, and SL planned the study and approved the initial study protocol. DO took the lead in drafting the manuscript along with significant contributions from AM, HL, and EH. All authors provided critical feedback and helped to shape the final draft of the manuscript. Addressed on manuscript page 15.  Name and contact information for the trial sponsor.  Indiana University. Human Subjects and IRB contact information: [irb@iu.edu](mailto:irb@iu.edu), (317) 274-8289. Addressed on manuscript page 1. |
|  | 5c | Role of study sponsor and funders, if any, in study design; collection, management, analysis, and interpretation of data; writing of the report; and the decision to submit the report for publication, including whether they will have ultimate authority over any of these activities.  This funding source and sponsor had no role in the design of this study and will not have any role during its execution, analysis, interpretation of the data, or decision to submit results. Addressed on manuscript page 15. |
|  | 5d | Composition, roles, and responsibilities of the coordinating centre, steering committee, endpoint adjudication committee, data management team, and other individuals or groups overseeing the trial, if applicable (see Item 21a for data monitoring committee).  Research Operations Committee: Principal Investigator, trauma surgeons, geriatrician, research coordinator, research assistants, and analyst meet weekly. Review of weekly enrollments and workflow, oversight of publication of study reports, preparation of protocol and revisions, and preparation of presentations of study findings. Addressed on manuscript page 12. |
| Introduction |  |  |
|  |  |  |
| Background and rationale | 6a | Description of research question and justification for undertaking the trial, including summary of relevant studies (published and unpublished) examining benefits and harms for each intervention.  Introduction: According to the United States Census Bureau, the estimated population in the United States in 2018 aged 65 years old or greater is 52,443,114 people. From 2014-2060 the age group from 45-64 is expected to increase 19.8% and for those over 65, the increase is projected to be 112%.^1^ Injury from motor vehicle crashes, falls, gunshot wounds, stabs, and natural or man-made disasters lead to 1.4 million hospitalizations per year in persons age 50 or older, and billions in healthcare costs.^2,3^ In addition to monetary costs is the emotional toll that weighs on patients and their loved ones, inhibiting their ability to fully recover. The prevalence of post-traumatic stress disorder (PTSD) in this vulnerable population of Americans varies from 40% at discharge to 23% at one year after injury. Similarly, up to 35% of trauma survivors demonstrate symptoms of depression.^4-9,10-12^ Addressed on manuscript page 2.  Existing Knowledge: For patients without traumatic brain injuries or spinal cord injuries, the non-neurologically injured, there is the potential for near full recovery from both physical and psychological disabilities and a return to a pre-injury level quality of life. However, the current health care system is fragmented, and these vulnerable patients are often left with residual physical and psychological disabilities and reduced quality of life for years after injury. Many leave the hospital without an established primary care provider or with a failure to communicate the recovery plans to an existing primary care provider. According to the Institute of Medicine (IOM), post injury care should be evidence based, patient-centered, without fragmentation, and situated within a collaborative care model. Recent randomized controlled trials have found that the collaborative care model was able to enhance quality of life, reduce care fragmentation, and improve psychiatric symptoms among traumatic brain or spinal cord injury survivors.^15-18^ However, there has been no similar intervention targeting the non-neurologically injured patient. Collaborative care models have been successfully employed at our institution as demonstrated in the GRACE and IMPACT studies.^19-20^ Addressed on manuscript page 2.  Need for a trial: This randomized controlled clinical trial aims to test the efficacy of a collaborative care model, named the Trauma Medical Home (TMH), in meeting the complex biophysical recovery needs of injured older adults without neurologic injury. The findings of this trial will provide trauma centers and trauma systems nationwide with a collaborative care model that can be replicated and implemented to improve the lives of older injured patients. Addressed on manuscript pages 2 and 3. |
|  | 6b | Explanation for choice of comparators.  The primary intervention of this study is the TMH collaborative care model, which will use evidence-based protocols and an interdisciplinary team approach to address specific rehabilitation needs of a non-neurologically injured older adult. Collaborative care models have previously been shown to improve patient outcomes and decrease fragmentation in care. Upon enrollment and informed consent by an RA/RC, participants will have a baseline assessment. They will then be randomized to usual care or intervention by an unblinded research staff. All participants receive usual care. Patients randomized to the intervention will also receive the TMH collaborative care intervention. After the baseline assessment, they will also set up a time for the First Home Visit. There are five phases to the intervention, followed by Outcomes Assessments at 6- and 12-months. The intervention is intentionally timed during the most vulnerable phases of the conceptual recovery model.  Usual care refers to the current practice of discharging an injured patient with follow-up care provided at the discretion of various specialists, depending on injury types, without a formal plan for coordination of specialists. There is also no standardized format to assess psychological well-being after discharge. Upon discharge from acute care, patients are provided with a discharge summary and discharge instructions describing the patient’s hospital course, injuries, new diagnoses, medications with dosages, and post-injury rehabilitation plan. Patients may also receive educational materials on caregiver coping mechanisms, and legal and financial advice if requested. Patients are encouraged to follow-up with their primary care provider or injury specialist for any continuing care needs. There is no formalized system of coordinating appointments or care plans between specialist teams and the primary care provider. Patients in the usual care arm will receive no further interventions. Usual care is the current standard at most trauma hospitals.  Comparative effectiveness can then be assessed between usual care vs the intervention. Addressed on pages 5 and 6 of the manuscript and the potential impact is addressed on page 3 of the manuscript. |
| Objectives | 7 | Specific objectives or hypotheses  This randomized controlled clinical trial aims to test the efficacy of a collaborative care model, named the Trauma Medical Home (TMH), in meeting the complex biophysical recovery needs of injured older adults without neurologic injury. We hypothesize that individuals seen in the TMH for six months will report a higher quality of life and lower healthcare utilization compared to individuals receiving only usual care. The TMH intervention will primarily aim to investigate the 6- and 12-month physical function, psychological recovery and healthcare utilization in injured older adults. We hypothesize that coordinating care in a multi-disciplinary manner will improve physical and psychological outcomes and decrease healthcare utilization while increasing cost effectiveness. Addressed on manuscript pages 3 and 7. |
| Trial design | 8 | Description of trial design including type of trial (eg, parallel group, crossover, factorial, single group), allocation ratio, and framework (eg, superiority, equivalence, noninferiority, exploratory).  This is a prospective, single-blind multi-center, randomized controlled clinical trial utilizing a computer-generated stratified randomization scheme into the TMH intervention or control group (usual care) in a 1:1 manner. All participants will get usual care. Those randomized to the TMH intervention will *also* receive the collaborative care (TMH) intervention. Outcome assessments will be performed on each group by blinded research staff, and mixed-effects models will be used to determine superiority of the TMH intervention on physical, psychological and health utilization outcomes. Addressed on manuscript page 5. |
| Methods: Participants, interventions, and outcomes | | |
| Study setting | 9 | Description of study settings (eg, community clinic, academic hospital) and list of countries where data will be collected. Reference to where list of study sites can be obtained.  The study sites include Indiana University Health Methodist Hospital, Sidney & Lois Eskenazi Hospital, St. Vincent Hospital in Indianapolis, Indiana, and University of Wisconsin-Madison in Madison, Wisconsin. Combined, these trauma centers evaluate and treat well over 9,000 injured patients annually. Indiana University Health, Methodist Hospital has 625 beds, with 2,967 yearly trauma admissions, and Sidney & Lois Eskenazi Hospital has 327 beds and admits 1,645 trauma patients yearly. Methodist and University of Wisconsin are large academic centers and receive trauma transfers from across the Midwest. Eskenazi Health is a public access hospital serving the local community in Marion County. It is the largest safety-net hospital in the state of Indiana. St. Vincent has 616 beds and admits 2,852 trauma patients, and University of Wisconsin has 505 beds and admits almost 3,000 trauma patients annually. Addressed on manuscript page 3. |
| Eligibility criteria | 10 | Inclusion and exclusion criteria for participants. If applicable, eligibility criteria for study centres and individuals who will perform the interventions (eg, surgeons, psychotherapists). This study will be conducted in a population of English-speaking injured adults, age 50 and over, with an injury severity score (ISS) of 9 or greater without neurologic injury (brain or spinal cord injury) or pre-existing diagnosis of neurodegenerative disease (including dementia, Alzheimer disease, or Parkinson disease) that reside within 50 miles of the admitting trauma center and have access to a telephone. Eligible individuals must be able to provide informed consent, or have a legally authorized representative provide informed consent. Exclusion criteria are defined by the presence of: significant head injury (defined as any intracranial blood on Computed Tomography scan of the head, or Glasgow Coma Scale score of less than 13), a burn that involves greater than 10% of total body surface area, pregnancy (determined by a urine pregnancy test), incarceration, acute stroke upon admission or while hospitalized, malignancy with less than one-year of life expectancy, recent alcohol or drug use disorder(within the past 6 months) as determined from the medical record and/or the Drug Abuse Screening Test or the Alcohol Use Disorders Screening Test C (Audit-C), or sensory impairment that would preclude active participation with study assessments and/or communications. Addressed on manuscript page 4. |
| Interventions | 11a | Interventions for each group with sufficient detail to allow replication, including how and when they will be administered.  The primary intervention of this study is the TMH collaborative care model, which will use evidence-based protocols and an interdisciplinary team approach to address specific rehabilitation needs of a non-neurologically injured older adult. Upon enrollment and informed consent by an RA/RC, participants will undergo the baseline assessment. They will then be randomized to usual care or intervention by an unblinded research staff member. If randomized to the intervention, they still receive usual care, and they will also set up a time for the First Home Visit by a trained Collaborative Care Nurse (CCN) prior to or shortly after discharge from acute care. Information collected at the First Home Visit will trigger the use of specific evidence-based care protocols and the interdisciplinary team will work together weekly to develop a personalized plan of care that will be implemented over the 6-month intervention period. The CCN will take the care plan back to the patient and work with them throughout the intervention on a mutually agreed upon schedule. Using a mobile office concept, the patient or caregiver can decide the most convenient follow-up method, whether it be meeting at their home, at a physician office, place in the community, or via phone interaction. The Healthy Aging Brain Care Monitor (HABC-M) will be used throughout the course of the intervention to dynamically inform the activation and deactivation of care protocols to best suit patients’ changing needs. Addressed on manuscript pages 5 and 6, and Table 1 on pages 19-21. |
|  | 11b | Criteria for discontinuing or modifying allocated interventions for a given trial participant (eg, drug dose change in response to harms, participant request, or improving/worsening disease).  Participants or their surrogates can choose to leave the study at any time, for any reason. In the rare case of an adverse event from the intervention, this will be reported to the data safety monitoring board and reviewed for appropriate response. Addressed on manuscript page 4 and 5. |
|  | 11c | Strategies to improve adherence to intervention protocols, and any procedures for monitoring adherence (eg, drug tablet return, laboratory tests). :  The Trauma service census will be screened daily by the research coordinator for eligible participants. Eligible participants will be approached prior to hospital discharge and provided information on the study.  Gift card incentives will be utilized at the completion of the 6- and 12-month follow up visits.  The CCN’s mobile office concept also helps to improve adherence to follow-up. The patient and/or caregiver identifies the most convenient location for follow-up meetings. This could be at home, at the clinic or hospital, or designated areas in the community. The TMH validated care protocols address various issues such as medication adherence and compliance with physical therapy.  Additionally, the interactive phase stresses the importance of frequent communication between the patient and caregiver and the care coordinator, which can be face-to-face, by telephone, or by electronic means. Patients must have access to a telephone to be participants in the study.  Study retention will be assessed periodically. If retention drops below 80%, the study staff will follow-up with the study participants to troubleshoot issues and provide coaching in order to prevent more losses. Addressed in manuscript page 12-13. |
|  | 11d | Relevant concomitant care and interventions that are permitted or prohibited during the trial.  All usual concomitant care is permitted as long as the patient does not meet any of the exclusion criteria. Addressed on manuscript page 6. |
| Outcomes | 12 | Primary, secondary, and other outcomes, including the specific measurement variable (eg, systolic blood pressure), analysis metric (eg, change from baseline, final value, time to event), method of aggregation (eg, median, proportion), and time point for each outcome. Explanation of the clinical relevance of chosen efficacy and harm outcomes is strongly recommended.  The following four instruments will be used to assess participant outcomes.  **Short Physical Performance Battery (SPPB):** Physical recovery effects will be assessed via the SPPB, a validated objective assessment. The SPPB yields a performance score of 0-12; 0-4 poor, 5-7 intermediate, 8-12 is good. Based on previous studies in similar patient populations the expected scores on the SPPB are 6.0 (SD 2.5) (at baseline), 7.5 (SD 2.5) at 6-months, and 8 (SD 2.5) at 12 months.  A difference of more than 1.3 would be considered clinically significant between the control and intervention groups.  **Medical Outcome Study Short Form (SF-36):** Non-neurologically injured patient’s health–related quality of life will be assessed using the Medical Outcome Study Short Form (SF-36). This scale has eight components (physical functioning, role-physical, bodily pain, general health, vitality, social functioning, role-emotional, and mental health) that are aggregated into a Physical Component Summary (PCS) and a Mental Component Summary (MCS).​ Expected scores on the PCS of the SF-36 are 40 (SD 5) at baseline, 49 (SD 5) at 6-months, and 51 (SD 5) at 12-months.  A difference of more than 2 would be considered clinically significant for both the PCS and MCS.  **Patient Health Questionnaire-9 (PHQ-9) and Generalized Anxiety Disorder Scale (GAD-7):** The PHQ-9 is a nine-item depression scale with a total score from 0 to 27 and the GAD-7 is a seven-item anxiety scale with a total score from 0 to 21. Both of these scales are derived from the Patient Health Questionnaire, have good internal consistency, and test–retest reliability as well as convergent, construct, criterion, procedural and factorial validity for the diagnosis of major depression and general anxiety disorder.  **Health Care Utilization and Cost-effectiveness**: We will use the Eskenazi Health financial record system and the local data-warehouse of Indiana University Health (IUH) and IUH Physician Group(IUHP) to capture data needed to determine health care utilization. The IUHP data warehouse includes detailed administrative, billing and hospital records of all patients seen within the IUH system, which encompasses nearly 60% of health care in the state of Indiana. Furthermore, we will use the data from the Indiana Network for Patient Care (INPC) to capture any health care utilization outside of the IUH system. INPC is the primary health information exchange in the state of Indiana and it provides data for acute care services from all of the health care systems within the state. We will determine the number of emergency department visits and the number of re-hospitalizations throughout the entire study period as well as the diagnoses associated with each utilization episode.  Addressed on manuscript pages 7, 8, 9, and 10. |
| Participant timeline | 13 | Time schedule of enrolment, interventions (including any run-ins and washouts), assessments, and visits for participants. A schematic diagram is highly recommended  See Table 1 and Table 2 on manuscript pages 19, 20, and 21. |
| Sample size | 14 | Estimated number of participants needed to achieve study objectives and how it was determined, including clinical and statistical assumptions supporting any sample size calculations.  This randomized controlled trial recruiting a sample size of 430 patients (215 per group) is powered to detect a significant effect of 0.325 standard deviation or larger with 80% power on our outcome measurements. This is anticipating 30% lost to follow-up or death at the 12-month assessment. This sample size estimation is based on previous literature using the SPPB score.^12,21,39-41^ Assuming a mean SPPB score of 6.0 (SD 2.5), a sample size of 150 is needed to obtain 80% power and to detect a change score of 0.81, using a two-sample t-test at 0.05. Addressed on manuscript page 3-4. |
| Recruitment | 15 | Strategies for achieving adequate participant enrolment to reach target sample size.  The Trauma service census will be screened daily by the research coordinator for eligible participants at each site. All trauma admissions are reviewed for study eligibility. Participation across sites may vary based on patient demographics and injury characteristics. Eligible participants will be approached prior to hospital discharge and provided information on the study. If the potential participant is interested in study participation, full informed consent will take place either in-hospital, or following discharge. Addressed on manuscript page 12-13. |
| **Methods: Assignment of interventions (for controlled trials)** | | |
| Allocation: |  |  |
| Sequence generation | 16a | Method of generating the allocation sequence (eg, computer-generated random numbers), and list of any factors for stratification. To reduce predictability of a random sequence, details of any planned restriction (eg, blocking) should be provided in a separate document that is unavailable to those who enrol participants or assign interventions.  A computer-generated stratified blocked randomization scheme will be used to generate an allocation sequence. Randomization is stratified by injury severity score (ISS), mechanism of injury and recruitment site. Random block sizes of 2 and 4 are used within each strata. Addressed on manuscript page 6-7. |
| Allocation concealment mechanism | 16b | Mechanism of implementing the allocation sequence (eg, central telephone; sequentially numbered, opaque, sealed envelopes), describing any steps to conceal the sequence until interventions are assigned. Following eligibility confirmation and informed consent, a separate unblinded research staff member will implement the computerized allocation sequence.  The RA’s who collect outcome assessments are different than those who recruit patients. Another independent RA makes the CCN aware of the subjects who are randomized to the intervention arm. These various staff members do not overlap in their responsibilities. Addressed on manuscript page 6-7. |
| Implementation | 16c | Who will generate the allocation sequence, who will enrol participants, and who will assign participants to interventions.  The allocation sequence is computer-generated. The RC and RA will enroll participants. A separate RA will assign participants to an intervention based on a computer-generated allocation sequence. Addressed on manuscript page 6-7. |
| Blinding (masking) | 17a | Who will be blinded after assignment to interventions (eg, trial participants, care providers, outcome assessors, data analysts), and how.  The RC and RA will be blinded to the treatment assignment. A separate unblinded RA will randomize patients after the RC and RA have completed eligibility confirmation and baseline assessments. The CCN is unblinded and is notified of the patients assigned to the intervention arm. Trial participants and care providers must be unblinded due to the nature of the intervention. Addressed on manuscript page 7. |
|  | 17b | If blinded, circumstances under which unblinding is permissible, and procedure for revealing a participant’s allocated intervention during the trial.  We do not anticipate the need for unblinding during the trial, as the risk of harm to the participants is so low. However, if at any time there is a concern about the need for unblinding, this will be reviewed by the Research Operations Committee and the IRB for proper procedure. Addressed on manuscript page 12. |
| **Methods: Data collection, management, and analysis** | | |
| Data collection methods | 18a | Plans for assessment and collection of outcome, baseline, and other trial data, including any related processes to promote data quality (eg, duplicate measurements, training of assessors) and a description of study instruments (eg, questionnaires, laboratory tests) along with their reliability and validity, if known. Reference to where data collection forms can be found, if not in the protocol.  Demographic and medical data will be collected at the baseline assessment and at follow-up assessments, including the subject’s age, race, gender, years of education completed, income bracket, height, weight, body mass index, blood pressure, and Charlson Comorbidity Index. We will also utilize the trauma registries maintained at the trauma centers to obtain detailed demographics, injury type, injury location, injury severity, treatment and complication information.  We will use the Eskenazi Health financial record system and the local data-warehouse of Indiana University Health (IUH) and IUH Physician Group(IUHP) to capture data needed to determine health care utilization. The IUHP data warehouse includes detailed administrative, billing and hospital records of all patients seen within the IUH system, which encompasses nearly 60% of health care in the state of Indiana. Furthermore, we will use the data from the Indiana Network for Patient Care (INPC) to capture any health care utilization outside of the IUH system. INPC is the primary health information exchange in the state of Indiana and it provides data for acute care services from all of the health care systems within the state. We will determine the number of emergency department visits and the number of re-hospitalizations throughout the entire study period as well as the diagnoses associated with each utilization episode.  All baseline assessments will be completed in the participant’s hospital room by trained and blinded RC/RA. Follow-up assessments will be completed by the RC/RA’s at a mutually agreed upon location with the participant or their proxy. Assessments will be entered into a REDCap (Research Electronic Data Capture) database, an electronic data capture tool hosted at Indiana University Clinical Translational Science Institute. REDCap is a secure, web-based application designed to support data capture for research studies, providing validated data entry, audit for tracking data manipulation and export procedures, automated export procedures and procedures for importing data from external sources. Frequency, timing of contacts and the intervention offered in the group receiving TMH will be tracked using the HABC Trauma Medical Home software which offers quantitative measures of intervention intensity in the TMH group. All of the cognitive, physical, psychological, and quality of life outcome measures will be assessed at baseline (hospital discharge), and at 6- and 12-month follow-up. While some assessments are recorded on paper, all are directly entered into the electronic system in REDCap. To monitor data quality and completeness, a separate RA or RC than the assessor reviews the data entered within a week of entry.  Addressed on manuscript page 8-9. |
|  | 18b | Plans to promote participant retention and complete follow-up, including list of any outcome data to be collected for participants who discontinue or deviate from intervention protocols.  Gift card incentives will be utilized at the completion of the 6- and 12-month follow up visits. Additionally, the interactive phase stresses the importance of frequent communication between the patient and caregiver and the care coordinator, which can be face-to-face, by telephone, or by electronic means. Patients must have access to a telephone to be participants in the study.  Study retention will be assessed periodically. If retention drops below 80%, the study staff will follow-up with the study participants to troubleshoot issues and provide coaching in order to prevent more losses.  All data collected up to the point of discontinuation will be used for analysis. Addressed on manuscript page 12-13. |
| Data management | 19 | Plans for data entry, coding, security, and storage, including any related processes to promote data quality (eg, double data entry; range checks for data values). Reference to where details of data management procedures can be found, if not in the protocol.  All consented participants are assigned a unique study identifier upon study enrollment. Only IRB-approved study team members have access to the collected data. The data is stored in a secure REDCap (Research Electronic Data Capture) database on a password protected IU server. REDCap was specifically developed around HIPAA security guidelines. To monitor data quality and completeness, a separate RA or RC than the assessor reviews the data entered within a week of entry.Addressed on manuscript page 4-5 and 9. |
| Statistical methods | 20a | Statistical methods for analysing primary and secondary outcomes. Reference to where other details of the statistical analysis plan can be found, if not in the protocol.  Participant baseline characteristics will be compared using analysis of covariance (ANCOVA) for continuous variables and the Cochran-Mantel-Hansel statistic for categorical variables while controlling for recruitment sites to verify the comparability of the randomized groups. Data distributions and frequencies will be examined. Alternative approaches such as transformations or nonparametric methods will be used if continuous data does not follow normal distributions. In the case of zero or small cell sizes, we will adopt exact inference procedures. SAS 9.4 (SAS Institute, Cary, North Carolina) will be used for all analysis and significance noted at p<0.05.  Mixed effect models will be used to evaluate the physical function scores (SPPB, PCS on the SF-36) collected at baseline, 6- and 12-months. Independent variables will include the assigned group, time of evaluation, and a group and time interaction while adjusting for stratification variables (recruitment site, injury severity, injury type) and baseline covariates that are found to be significantly different between the intervention and usual care group. To account for the potential correlations between the physical function scores (SPPB, PCS on the SF-36) within individuals over time, an unstructured variance-covariance matrix will be used in the mixed effects model. Following significant interactions between group and time, post-hoc comparisons will be conducted at each follow-up time to determine the point at which a group difference is detectable. A maximum likelihood approach will be used to generate parameter estimates and inferences, which are robust under several missing data mechanisms.  The mixed effect modeling approach outlined for our primary outcome will be applied to investigate our secondary outcome of psychological recovery measured with PHQ-9, GAD-7, and MCS on SF-36 scores collected at baseline, 6- and 12-months.  To examine the ability of the TMH intervention to reduce health care utilization, a Cox’s proportional hazard model will be used. Event time will be censored at 12-months for those participants who are followed to 12-months without experiencing any outcome event. Participants who died or were lost to follow-up will have their observation time censored at time of death or data of last contact. The outcome is measured as time from enrollment to emergency department visits and hospital readmissions. Group assignment, time of evaluation, and baseline covariates found to differ significantly between groups will be included in the model.  The economic value and costs associated with the TMH intervention will be evaluated using an established method from a Medicare payment perspective. The cost effectiveness of the intervention is measured by increments in the cost summary (both health care and non-health care related expenses) and effect (return to function measured by SF-36). Foregone economic opportunities are captured by the recovery time in dollar value. Multivariate regression models will examine total health care costs in the 12-month post index period. These data will inform the cost-effectiveness ratio, which is the difference in intervention costs of the treatment arms, divided by the difference in effectiveness between groups. Sensitivity analysis and bootstrapping will be completed to ensure robustness of findings.  Addressed on manuscript pages 10-11. |
|  | 20b | Methods for any additional analyses (eg, subgroup and adjusted analyses).  Not applicable. There are no planned additional analyses. |
|  | 20c | Definition of analysis population relating to protocol non-adherence (eg, as randomised analysis), and any statistical methods to handle missing data (eg, multiple imputation).  Protocol non-adherence will be evaluated using an intention to treat analysis. Two different forms of missing data are anticipated with this trial; those lost to follow-up and those lost to death. The usual care group may experience a higher rate of loss when compared to the trial group due to frequent contacts between the study team and the participants in the intervention group. In anticipation of these missing data, we will use a missed effects model approach, which is robust under the missing at random assumption, i.e. the probability of missing is unrelated to the missing outcomes. Baseline characteristics of patients with missing outcomes will be compared to detect potential violations to the missing at random assumption. Sensitivity analyses using various methods of imputation or a full parametric likelihood approach assuming various patterns of missing data will be performed if the missing at random assumption is violated. Addressed on manuscript page 12. |
| **Methods: Monitoring** | | |
| Data monitoring | 21a | Composition of data monitoring committee (DMC); summary of its role and reporting structure; statement of whether it is independent from the sponsor and competing interests; and reference to where further details about its charter can be found, if not in the protocol. Alternatively, an explanation of why a DMC is not needed  The Data Safety and Monitoring Board (DSMB) meets every six months and is composed of three members; a safety officer, an expert trauma surgeon, and a biostatistician. This team is independent from IU and has no competing interests. The safety officer is a physician researcher with extensive experience in intervention studies. This individual reviews reports generated by the study manager and biostatistician to determine if additional action is necessary. This additional action could include corrective action, an ad hoc review, a stopping rule violation, and the need to communicate out of range data to the provider or patient. All reports will be provided to the IRB at the time of continuing review. Addressed on manuscript page 11-12.  DSMB main contact: Dr. Marcel Salive, [marcel.salive@nih.gov](mailto:marcel.salive@nih.gov).  Additional DSMB members:  [Londamody@med.umich.edu](mailto:Londamody@med.umich.edu)  [Christopher.cox@duke.edu](mailto:Christopher.cox@duke.edu)  Oscar.guillamondegui@vumc.org |
|  | 21b | Description of any interim analyses and stopping guidelines, including who will have access to these interim results and make the final decision to terminate the trial.  Data will be evaluated by the DSMB every 6 months and will review participant accrual, drop out rates, adverse events, participant complaints, compliance to interventions, protocol violations or noncompliance, out of range data, risk-benefit ratio assessment, and stopping rules report. These interim results will be made available to the IRB. Ultimately the final decision to terminate the trial will be up to the IU IRB. Addressed on manuscript page 11-12 and Table 3 on manuscript page 22. |
| Harms | 22 | Plans for collecting, assessing, reporting, and managing solicited and spontaneously reported adverse events and other unintended effects of trial interventions or trial conduct.  Collaborative care interventions are associated with a low adverse event rate. Nonetheless, each adverse event occurrence will generate a blind report that will be reviewed immediately by the PI and the biostatistician, and every 6 months by the DSMB. This study will follow the Indiana University IRB policy for the reporting of adverse events. The study staff will use an adverse event form to report injuries or other adverse events caused by the intervention. Research staff routinely monitor the EMR for any new ED visits or hospitalizations. These are recorded on an electronic spreadsheet. Deaths are also reported either by a notification in the EMR or from family members when they are called to schedule Outcomes Assessments. These reports are provided to the DSMB at each meeting. Other adverse events would be provided by spontaneous reports from patients or their families. Addressed on manuscript page 5. |
| Auditing | 23 | Frequency and procedures for auditing trial conduct, if any, and whether the process will be independent from investigators and the sponsor.  The trial will be audited every 6 months by the DSMB and reviewed annually by the Indiana University IRB through the continuing review. Addressed on manuscript page 11-12. |
| Ethics and dissemination | | |
| Research ethics approval | 24 | Plans for seeking research ethics committee/institutional review board (REC/IRB) approval.  The Indiana University Institutional Review Board approved this randomized control trial to test the effectiveness of the TMH intervention (IRB#1612690852) and is listed on clinictrials.gov (NCT03108820). Addressed on manuscript pages 1 and 4. |
| Protocol amendments | 25 | Plans for communicating important protocol modifications (eg, changes to eligibility criteria, outcomes, analyses) to relevant parties (eg, investigators, REC/IRBs, trial participants, trial registries, journals, regulators).  In the event of protocol amendments, the Indiana University IRB will be notified immediately and a change of protocol request will be submitted for approval. Upon IRB approval, the PI will inform the relevant parties as needed through formal communications and the weekly Research Operations Committee meeting. Addressed on manuscript page 12. |
| Consent or assent | 26a | Who will obtain informed consent or assent from potential trial participants or authorised surrogates, and how (see Item 32).  Informed consent will be obtained by a trained Research Assistant (RA) or Research Coordinator (RC) upon enrollment. Informed consent may be obtained from a legally authorized representative if the participant is unable to provide consent. Any concerns about the patient’s capacity to provide informed consent is brought to the attention of a trauma physician. Informed consent will be obtained through a printed document that will be provided to the trial participant, as well as through a verbal consenting process. Copies of the signed consent documents will be provided to the trial participant or proxy upon completion. Addressed on manuscript page 4. |
|  | 26b | Additional consent provisions for collection and use of participant data and biological specimens in ancillary studies, if applicable.  Biological specimens will not be collected. A signed consent must be obtained from every participant in an ancillary study if the data collection/request is not covered in the original informed consent process. Any ancillary studies will go through IRB review. Addressed on manuscript page 4. |
| Confidentiality | 27 | How personal information about potential and enrolled participants will be collected, shared, and maintained in order to protect confidentiality before, during, and after the trial.  All research personnel will be IRB approved and appropriately trained on confidentiality procedures. Potential and enrolled participant data will be collected, shared, and maintained in accordance with the IU IRB and HIPAA guidelines. Addressed on manuscript page 4 and 5. |
| Declaration of interests | 28 | Financial and other competing interests for principal investigators for the overall trial and each study site.  We have no financial or competing interests to declare. Addressed on manuscript page 15. |
| Access to data | 29 | Statement of who will have access to the final trial dataset, and disclosure of contractual agreements that limit such access for investigators.  Not applicable. There are no contractual agreements that would limit access to investigators. |
| Ancillary and post-trial care | 30 | Provisions, if any, for ancillary and post-trial care, and for compensation to those who suffer harm from trial participation.  There is a very low risk of harm from trial participation, and if such issues arise, will be reviewed on a case by case basis. Post-trial care will continue as per the patient’s physicians. Addressed on pages 5 and 6, and Table 1 on pages 19-21. |
| Dissemination policy | 31a | Plans for investigators and sponsor to communicate trial results to participants, healthcare professionals, the public, and other relevant groups (eg, via publication, reporting in results databases, or other data sharing arrangements), including any publication restrictions.  The investigators plan to publish the results in abstracts and papers submitted to academic journals, as well as to disseminate these results at medical conferences (e.g. American Association for the Surgery of Trauma (AAST) Annual Meeting). Addressed on manuscript page 12. |
|  | 31b | Authorship eligibility guidelines and any intended use of professional writers.  No professional writers will be used. Substantive contributions to the protocol design and manuscript writing and revision are recognized for authorship in the manuscript. Addressed on manuscript page 15. |
|  | 31c | Plans, if any, for granting public access to the full protocol, participant-level dataset, and statistical code.  There are currently no plans for granting public access to the dataset and statistical code. |
| Appendices |  |  |
| Informed consent materials | 32 | Model consent form and other related documentation given to participants and authorised surrogates.  A copy of the trial consent form will be provided to participants or their surrogate. Addressed on manuscript page 4. Please see accompanying supplemental form for model consent form. |
| Biological specimens | 33 | Plans for collection, laboratory evaluation, and storage of biological specimens for genetic or molecular analysis in the current trial and for future use in ancillary studies, if applicable.  Not applicable. There will be no biological specimens collected. |

SPIRIT Figure

|  | **STUDY PERIOD** | | | | | | |
| --- | --- | --- | --- | --- | --- | --- | --- |
|  | **Enrolment** | **Allocation** | **Post-allocation** | | | **Close-out** | |
| **TIMEPOINT** | ***Prior to or at Discharge*** | **Prior to or at Discharge** | ***1 mo*** | ***3 mo*** | ***6 mo*** | ***12 mo*** | |
| **ENROLMENT:** |  |  |  |  |  |  |  |
| **Eligibility screen** | X |  |  |  |  |  |  |
| **Informed consent** | X |  |  |  |  |  |  |
| **Allocation** |  | X |  |  |  |  |  |
| **INTERVENTIONS:** |  |  |  |  |  |  |  |
| **Usual Care** |  |  |  |  |  |  |  |
| **Trauma Medical Home** |  |  |  |  |  |  |  |
| **ASSESSMENTS:** |  |  |  |  |  |  |  |
| **SPPB** | X |  |  |  | X | X |  |
| **PHQ-9** | X |  |  |  | X | X |  |
| **GAD-7** | X |  |  |  | X | X |  |
| **SF-36** | X |  |  |  | X | X |  |

*It is strongly recommended that this checklist be read in conjunction with the SPIRIT 2013 Explanation & Elaboration for important clarification on the items. Amendments to the protocol should be tracked and dated. The SPIRIT checklist is copyrighted by the SPIRIT Group under the Creative Commons “[Attribution-NonCommercial-NoDerivs 3.0 Unported](http://www.creativecommons.org/licenses/by-nc-nd/3.0/)” license.
