# Supplementary material for: A trauma medical home, evaluating collaborative care for the older injured patient: study protocol for a randomized controlled trial
Source: Trials. 2020 Jul 16;21:655. doi: 10.1186/s13063-020-04582-x (PMC7364470; doi:10.1186/s13063-020-04582-x)
Supplement: Supplementary file 2 — Additional file 2. Indiana University informed consent statement for elderly trauma medical home. [file 13063_2020_4582_MOESM2_ESM.pdf]

# INDIANA UNIVERSITY INFORMED CONSENT STATEMENT FOR

## Elderly Trauma Medical Home

You are invited to participate in a research study of physical and psychological recovery of injury survivors. You were selected as a possible subject because you suffered an injury and were admitted to the trauma center at the hospital. We ask that you read this form and ask any questions you may have before agreeing to be in the study.

The study is being conducted by the trauma surgeons at the Indiana University Department of Surgery as well as members of the Department of Medicine.

## STUDY PURPOSE

The purpose of this study is to determine if a type of intervention that incorporates multiple aspects of recovery can be used as a "recovery model" in the future to help older subjects recuperate both physically and mentally or at least help to slow down or minimize any long term effects from the injury.

## NUMBER OF PEOPLE TAKING PART IN THE STUDY

If you agree to participate, you will be one of up to 430 subjects who will be participating in this research. Your participation will last for 1 year.

## PROCEDURES FOR THE STUDY

If you agree to be in the study, the following things will happen:

**IN THE HOSPITAL:**
While you are in the hospital, we will get some information from your medical records regarding your injury, your medical history, and administer some questionnaires about your quality of life, your mood and anxiety level, possible depression. Before you are discharged from the hospital, we will ask you to provide the name of your primary care physician. If you do not have a primary care physician, we will work with you to help identify a physician for you. We will send a letter to your primary care provider that summarizes your injuries, what took place while you were in the hospital, the medications you are taking, and the plan for your rehabilitation and care. You will also receive some education on communication, as well as legal and financial advice. We will also provide some information on coping skills for your caregiver. All study participants will receive this part of the intervention.

**RANDOMIZATION**:
If you agree to participate in this study you will be randomly placed in one of two groups and will receive either the in-hospital portion of the intervention only, which is considered the current standard of care, or you will receive a personalized recovery program that continues after you leave the hospital. This recovery program begins with the first home visit described below. Randomization



Protocol 1612690852 IRB Approved

means that you will have an equal chance of being in the recovery program group or the standard care group.

**FIRST HOME VISIT:**

If you are assigned to the recovery program group, the care coordinator will complete a pre-home visit review and come to your house and conduct a face-to-face initial assessment of your residence. At your home, the care coordinator will conduct a cognitive and psychological assessment, evaluate any social and community needs you and your informal caregiver may have, and will review all prescribed and over the counter medications you are taking. You will be asked to perform some physical activities in order to assess your recovery progress, such as testing your strength and balance. If your injury does not allow you to stand or perform other parts of the test, the test can be changed to accommodate your needs. The care coordinator will also make note of all scheduled and recommended appointments with specialists, physical therapists and occupational therapists.

The care coordinator will work with the study team and your primary care provider to finalize your care plan. If needed, the care coordinator may refer to you for more extensive cognitive or psychological testing at local mental health practices.

The care coordinator will use a type of questionnaire called the “Healthy Aging Brain Care Monitor” (HABC-M) to monitor your cognitive, functional, and psychological symptoms and the stress of your caregiver. This tool will help us determine the best way to help you get better.

The care coordinator will also schedule a second home visit with you and your informal caregiver, which will take place about 1 to 2 weeks after this first home visit.

**SECOND HOME VISIT:**

During the second home visit, the care coordinator will review your recovery care plan with both you and your informal caregiver. This process will include discussions so that everyone understands how your recovery will be monitored and to make sure the appropriate recovery plans are implemented. Both you and your informal caregiver will receive educational recovery handouts, and we will make sure you are connected to in-home services and community resources, as needed.

**THE 6-MONTH RECOVERY PROGRAM PERIOD:**

Follow-up after the first and second home visits includes a 6-month interaction period between you and/or your informal caregiver and the care coordinator, either through a face-to-face home or clinic visit, phone contact, email, fax, or mail. At the end of the 6 months, you will be transitioned to receive full care by your primary care physician. The minimum amount of contact with the study team during this 6-month interaction period will be every two weeks.

During these interactions, the care coordinator will answer any questions that arose from previous visits, collect your feedback (and that of your caregiver), review medications and discuss how you are doing with taking these medications as prescribed, review any specialist and/or therapy appointments



Protocol 1612690852 IRB Approved

that you had and whether or not you attended these appointments. You and your informal caregiver will complete some questionnaires, including the Healthy Aging Brain Care Monitor (HABC-M), to trigger the use of specific care recovery plans and to assist the informal caregiver's access to appropriate community resources. Throughout the duration of the 6-month recovery program, the study team will continue to work with you, your informal caregiver, and your primary care providers and specialists to monitor, implement, and revise your recovery care plan, as needed.

**STUDY INTERVIEWS:**

All study participants will have a baseline interview, a 6 month interview, and a 12 month interview. These interviews consist of questionnaires asking you about your health and how you feel about your health, and short physical assessment (if you are physically able). Your participation in the study will end after you complete the 12 month interview.

**RISKS OF TAKING PART IN THE STUDY:**

While on the study, the possible risks are:

(1) *Fatigue, anxiety, stress, or embarrassment during the interviews.* It is possible that you could feel embarrassed by some of the study questions. Our study staff members are trained to be alert and sensitive to signs of fatigue, embarrassment, and other symptoms and to take appropriate actions when they are present. You may skip any questions that you don't want to answer and can stop the interview at any point. The interviews can also be split into two sessions if you are tired or physically uncomfortable.

(2) *Falls and/or muscle stiffness and soreness from the physical assessment*. Based on previous studies, the most likely risk is a fall, and mild muscle stiffness and soreness associated with increased exercise. Moderate-intensity physical activities are associated with a very low risk of musculoskeletal complications. We expect that any soreness that may occur during the testing will subside within a few days and be unlikely to occur again. Participants with persistent or very severe soreness will be encouraged to contact their primary care provider. We will have an emergency plan in place to handle any emergencies that might occur.

(3) *Exposure of confidential information.* We make every effort to ensure all information collected about you remains secure and is accessible only by study staff members, but we cannot guarantee absolute confidentiality. The exceptions are if you are about to harm yourself or others if the law requires we make the information known. All key personnel involved with this study have successfully completed Indiana University training courses on the protection of human subjects in research. Each subject will be given a unique subject ID number and this unique identifier will be used to track your data (rather than using names or hospital or social security numbers). All records will be kept under lock with access only by study personnel.



Protocol 1612690852 IRB Approved

**BENEFITS OF TAKING PART IN THE STUDY:**

We hope that you will benefit from participating in this study by experiencing an improved physical and psychological recovery from your injuries. However, you may not experience any benefit. That is what this study is evaluating. We hope this study will provide beneficial information that will help other victims of traumatic injuries in the future.

**WILL I RECEIVE MY RESULTS?**

We may learn things about you from the study activities which could be important to your health or to your treatment. If this happens, this information will be provided to you. This study includes a depression screening and a member of the study team will notify you if your results indicate that you are at a high risk of depression. You may need to meet with professionals with expertise to help you learn more about your research results. The study team/study will not cover the costs of any follow-up consultations or actions.

**ALTERNATIVES TO TAKING PART IN THE STUDY:**

Instead of being in the study, you have the option to not participate in this study. You will still be treated with the standard care that is always provided to those who have experienced a traumatic injury.

**CONFIDENTIALITY**

Efforts will be made to keep your personal information confidential. We cannot guarantee absolute confidentiality. Your personal information may be disclosed if required by law. Your identity will be held in confidence in reports in which the study may be published. Your study information will be stored in a secure database called "REDCap".

Organizations that may inspect and/or copy your research records for quality assurance and data analysis include groups such as the study investigator and his/her research associates, the Indiana University Institutional Review Board or its designees, and (as allowed by law) state or federal agencies, specifically the Office for Human Research Protections (OHRP), and the National Institutes of Health (NIH), etc., who may need to access your medical and/or research records.

For the protection of your privacy, this research is covered by a Certificate of Confidentiality from the National Institutes of Health. The researchers may not disclose or use any information, documents, or specimens that could identify you in any civil, criminal, administrative, legislative, or other legal proceeding, unless you consent to it. Information, documents, or specimens protected by this Certificate may be disclosed to someone who is not connected with the research:



Protocol 1612690852 IRB Approved

(1) if there is a federal, state, or local law that requires disclosure (such as to report child abuse or communicable diseases);
(2) if you consent to the disclosure, including for your medical treatment;
(3) if it is used for other scientific research in a way that is allowed by the federal regulations that protect research subjects;
(4) for the purpose of auditing or program evaluation by the government or funding agency;

A Certificate of Confidentiality does not prevent you from voluntarily releasing information about yourself. If you want your research information released to an insurer, medical care provider, or any other person not connected with the research, you must provide consent to allow the researchers to release it.

A description of this clinical trial will be available on **ClinicalTrials.gov**, as required by U.S. law. This website will not include information that can identify you. At most, the website will include a summary of the results. You can search this website at any time.

**FUTURE USE OF DE-IDENTIFIED DATA**

Information collected from you for this study may be used for future research studies or shared with other researchers for future research. If this happens, information which could identify you will be removed before any information is shared. Since identifying information will be removed, we will not ask for your additional consent.

**COSTS:**

There is no cost to you for participating in this study. You and/or your insurance company will be responsible for all standard of care procedures, drugs, tests, etc. Visits to any surgeons or other doctors, including your primary care doctor, physical therapy, and medications associated with your injury or other health problems are considered standard of care. You will not be responsible for the costs of the care coordinator visits or the three (3) interview visits that occur during the study period.

**PAYMENT**

You will not receive payment for taking part in this study.

**CONTACTS FOR QUESTIONS OR PROBLEMS**

For questions about the study or a research-related injury, contact the researcher Dr. Ashley Meagher at 317-962-9231.

In the event of an emergency, you may call 317-962-2000 and ask for Dr. Meagher to be paged.



Protocol 1612690852 IRB Approved

For questions about your rights as a research participant or to discuss problems, complaints or concerns about a research study, or to obtain information, or offer input, please contact the IU Human Subjects Office at (317) 278-3458 or at irb@iu.edu.

**VOLUNTARY NATURE OF STUDY**

Taking part in this study is voluntary. You may choose not to take part or may leave the study at any time. Leaving the study will not result in any penalty or loss of benefits to which you are entitled. Your decision whether or not to participate in this study will not affect your current or future relations with IU Health Methodist Hospital or Eskenazi Hospital.

Your participation may be terminated by the investigator without regard to your consent if you do not follow the study procedures or if the study is terminated by the investigator for any reason.

You will be told about new information that may affect your health, welfare, or willingness to stay in the study.

**SUBJECT'S CONSENT**

In consideration of all of the above, I give my consent to participate in this research study.

I will be given a copy of this informed consent document to keep for my records. I agree to take part in this study.

**Subject's Printed Name:**________________________________________

**Signature of Subject**:__________________________________________**Date**:______________________________
(must be dated by the subject)

**Printed Name of Person Obtaining Consent:**______________________

**Signature of Person Obtaining Consent**:__________________________**Date**:______________________________



Protocol 1612690852 IRB Approved
